# Effect of Egyptian Leek Leaf Extract Supplementation on Productive and Economic Performance of Broilers

**DOI:** 10.3389/fvets.2020.584921

**Published:** 2020-11-05

**Authors:** Hanan S. Al-khalaifah, Mohamed E. Badawi, Reda M. Abd El-Aziz, Mohamed A. Ali, Anaam E. Omar

**Affiliations:** ^1^Environment and Life Sciences Research Center, Kuwait Institute for Scientific Research, Kuwait City, Kuwait; ^2^Department of Nutrition and Clinical Nutrition, Faculty of Veterinary Medicine, Zagazig University, Zagazig, Egypt; ^3^Department of Physiology, Faculty of Veterinary Medicine, Zagazig University, Zagazig, Egypt; ^4^Department of Animal Wealth Development, Faculty of Veterinary Medicine, Zagazig University, Zagazig, Egypt

**Keywords:** broiler, leek leaf extract, performance, biochemical parameter, economic

## Abstract

Antibiotic growth promoters have been used to improve growth and feed conversion in the poultry industry for a long time; however, they were banned because of several life-threatening side effects in animals, poultry, and humans. This work was carried out to investigate the effect of leek (*Allium ampeloprasum var. kurrat*) leaf extract (LLE) as a non-traditional growth promoter and feed additive on growth performance, carcass characteristics, serum biochemical parameters, and economic efficiency of broilers. Hubbard unsexed 1-day-old broilers (*n* = 250) were fed with diets supplemented with LLE for 42 days. The experimental chicks were randomly assigned to one of the five treatment groups varying in LLE quantity in diets: 0% (control), 0.05, 0.1, 0.15, and 0.2%, with five replicates per treatment (50 chicks/treatment or 10 chicks/replicate). Results showed that LLE supplementation improved (*P* < 0.05) different growth performance parameters. Furthermore, dietary LLE not only decreased serum total cholesterol, triglyceride, low-density lipoprotein, and glucose levels but also increased serum high-density lipoprotein level compared to the control diet. The weight percentages of dressing (*P* = 0.022) and liver (*P* = 0.041) showed a marked increase after the addition of LLE. Return, net profit, and collective efficiency measures were increased (*P* = 0.001) in all LLE groups compared with the control group. Broilers that fed on diets containing 0.2% LLE showed the highest growth and economic efficiency. It could be concluded that supplementation with LLE in broilers has growth-promoting effects, improved biochemical parameters, carcass quality, and promoted economic efficiency through maximizing both return and net profit.

## Introduction

The use of growth promoters in the poultry industry leads to the net increase in the weight of broilers without the use of any additional dietary supplements. Recently, the use of medicinal plants and their extracts in controlling poultry diseases and growth promotion has gained attention because of their cost-effective applications and minimal side effects (1). Although antibiotic growth promoters were used to improve growth and feed conversion for a large sum of time, they were banned because of several side effects reported in animals, poultry, and humans (2). Finding a cheap, safe, and acceptable alternative to replace antibiotics is desirable. Therefore, vegetables, herbs, and edible plants and/or their extracts have been researched as valid non-traditional growth promoters, feed additives, and immunostimulants. Leek (*Allium ampeloprasum var. kurrat*) is a biennial plant related to the genus Allium (family Alliaceae or Liliaceae), including onion and garlic (3). They are considered one of the oldest cultivated plants in Egypt and are consumed for food and/or medical purposes. Also, leek and its leaves are considered a potential source of several bioactive or health-promoting compounds, including a high number of phytonutrients. Their extracts have been studied for antimicrobial, antioxidant, cytotoxic (4), hypoglycemic (5), hypolipidemic (6), and hypercholesterolemic (7) effects.

Although the use of various herbal extracts as dietary supplements has been reported to improve the growth and protection of broilers against several diseases (8), the potential use of leek leaf extract has remained elusive. The current trial was delineated to investigate the improvement of productive performance, carcass characteristics, some serum biochemistry, and economic efficiency of broiler chickens by leek (*Allium ampeloprasum var. kurrat*) leaf extract (LLE) dietary supplementation.

## Materials and Methods

### Preparation and Analysis of Leek Leaf (LLE) Ethanolic Extract

Fresh leek leaves free from physical defects were collected from a local market. The collected sample was then dried at 40°C for 72 h in an air convection oven, ground, and passed through a 150-μm mesh sieve. The dried material (500 g) was then extracted with 2,000 mL of solvent (70% ethanol) at room temperature for 72 h. The extract was filtered through a filter paper (Whatman no. 1), and the extraction and filtration processes were repeated three times. The solvent was separated under vacuum at 40°C using a rotary evaporator (BUCHI-water bath-480, Germany). The extract was then freeze-dried by Thermo-Electron Corporation-Heto power dry LL300 Freeze Dryer and kept in dark-colored containers away from the light at −20°C until use, according to the previous reports (9). The total phenols, flavonoids, and radical scavenging activity of LLE were assessed by following the previously reported procedures (10).

Total phenols were estimated as gallic acid equivalents (mg GA/g dry extract) by using the Folin–Ciocalteu reagent. An aliquot of 0.5 mL LLE was mixed with 2.5 mL of Folin–Ciocalteu reagent (previously diluted with water 1:10, V/V) and 2 mL of NaHCO_3_ (7.5%). The absorbance was measured by using a spectrophotometer at 765 nm after incubation at 40°C for 15 min.

Flavonoids were evaluated as mg rutin equivalents. Aluminum chloride (0.5 mL, 2%) in methanol was mixed with the same volume of LLE. The absorbance was measured by using a spectrophotometer at 415 nm after 1-h incubation at room temperature.

### Animals and Experimental Design

This study was conducted at the Poultry Research Farm and was approved by the Committee of Animal Welfare and Research Ethics, Faculty of Veterinary Medicine, Zagazig University, Egypt. Unsexed 1-day-old Hubbard chicks (*n* = 250) weighing 40 ± 1 g were selected from a local hatchery. The chicks were randomly assigned to five experimental treatment groups: 0% (control treatment 1), 0.05% (treatment 2), 0.1% (treatment 3), 0.15% (treatment 4), and 0.2% (treatment 5) for 42 days with five replicates/treatment (50 chicks/treatment; 10 chicks/replicate). Each replica contained equal numbers of males and females. First, LLE was mixed well with other dietary feed additives (as mineral, vitamin mixture, and amino acids) and then mixed with other main dietary ingredients (as corn and SBM). Broilers were vaccinated against Gamboro and Newcastle diseases. Broilers were observed on a daily basis and checked for any syndromes without mortalities during the whole experiment. Broilers were kept in separate pens with a stocking density of 10 birds/m^2^ under suitable temperature and proper lighting conditions for 6 weeks of feeding. The isocaloric and isonitrogenous diets were prepared to fulfill the nutrient requirements of the Hubbards (11) and were given in a mash form. In brief, diet in the starter period (0–10 days) contained 23.02% crude protein (CP) and 3,035.75 kcal/kg diet metabolizable energy (ME); the grower period (11–22 days) contained 20.58% CP and 3,110.69 kcal/kg diet ME; and the finisher period (23–42 days) contained 19.07% CP and 3,184.79 kcal/kg diet ME, as shown in Table 1. Broilers were fed with water and diet *ad-libitum*. Diets were examined for dry matter (hot air oven at 105°C), CP (Kjeldahl method), crude fiber (Weende method), ash (muffle furnace at 600°C), and ether extract (Soxhlet apparatus) according to Official Methods of Analysis by AOAC (12). ME was estimated based on the NRC (13) prediction equation.

**Table 1:** Ingredients and composition of the experimental diets.

**Ingredients**	**Experimental diets**		
	**Starter diets (1–10 days)**	**Grower diets(11–22 days)**	**Finisher diets (23–42 days)**
Yellow corn	56.055	61.405	63.405
Soybean meal, 48%	32.60	28.64	29.00
Corn gluten, 60%	4.60	3.00	0.00
Soybean oil	1.92	2.5	3.70
Calcium carbonate	0.59	0.56	0.48
Ca. dibasic phosphate	2.73	2.49	2.08
Common salt	0.35	0.25	0.27
Sodium bicarbonate	0.25	0.25	0.20
Mineral mixture^**^	0.072	0.074	0.074
Vitamin mixture^*^	0.033	0.031	0.031
Choline chloride, 60%	0.07	0.06	0.05
L-Lysine, Hcl, 79.80%	0.35	0.33	0.26
L-Methionine, 100%	0.29	0.27	0.30
L-Threonine, 99%	0.09	0.09	0.10
Mycofix select^a^	0.05	0.05	0.05
**Calculated composition**			
ME, kcal/kg	3035.75	3110.69	3184.79
CP, %	23.02	20.58	19.07
EE, %	4.62	5.29	6.47
CF, %	2.56	2.30	2.53
Ca, %	0.96	0.88	0.76
AP, %	0.48	0.44	0.38
Lysine, %	1.41	1.27	1.20
Methionine, %	0.68	0.62	0.61
**Analyzed composition**			
DM, %	90.60	90.30	90.70
CP, %	22.25	19.30	18.15
EE, %	4.50	5.00	6.25
CF,%	2.36	2.17	2.30
Ash,%	3.60	3.55	3.57

Premix* per kg of diet.

* *Vitamin mixture: vitamin A, 1 500 IU; vitamin D3, 200 IU; vitamin E, 10 mg; vitamin K3, 0.5 mg; thiamine, 1.8 mg; riboflavin, 3.6 mg; D pantothenic acid, 10 mg; folic acid, 0.55 mg; pyridoxine, 3.5 mg; niacin, 35 mg; cobalamin, 0.01 mg; biotin, 0.15 mg*.

** *Mineral mixture: Fe, 80 mg; Cu, 8 mg; Mn, 60 mg; Zn, 40 mg; I, 0.35 mg; Se, 0.15 mg*.

a *Mycofix select is anti-mycotoxin from Anani Company, Egypt*.

*ME, metabolizable energy; DM, dry matter; CP, crude protein; EE, ether extract; CF, crude fiber; Ca, calcium; AP, available phosphorus*.

### Growth Performance Parameters

The individual chicks were weighed at the beginning and after 6-week feeding to obtain the live body weight (LBW). In a similar manner, body weight gain (BWG) was calculated as the final minus initial body weight. An average feed intake (FI, g/bird) was calculated as the difference between weights of the feed offered, and residues left after consumption by broilers and divided by the number of birds. The feed conversion ratio (FCR) was calculated according to previous reports (14).

### Carcass Traits

After 42 days of feeding, five broilers from each experimental treatment group (one from each replicate) were randomly selected, fasted overnight, weighed, and then slaughtered using a sharp knife to until complete bleeding. The dressing percentage in which the head, neck, feet, and lower wings were removed was estimated by final weighing, followed by the plucking of the feathers and evisceration. The liver, heart, stomach, intestine, and spleen were also weighed and expressed as a percent of LBW.

### Biochemical Analysis

The blood samples from five broilers per treatment group, one from each replicate, were collected after slaughtering in sterile glass tubes without anticoagulant and placed in a slanted position for 20 min at room temperature, followed by 10-min centrifugation at 3,000 rpm. The serum was then removed and stored at −20°C until further use for biochemical study by using various diagnostic kits (Roche Diagnostics, GmbH, USA). The total protein and albumin were estimated by using diagnostic kits described in the previous report (15), while the globulin was estimated by subtracting the albumin value from the total protein value. Alanine aminotransferase (ALT) (16) and serum aspartate-aminotransferase (AST) (17) were estimated according to the previously described methods. The Fossati (18) procedure was used to estimate the blood creatinine, urea nitrogen, and uric acids. Glucose (19), total cholesterol (20), triglyceride (21), HDL-cholesterol (22), and LDL cholesterol (23) were estimated according to the previously described methods.

### Determination of Economic Efficiency

Cost parameters were categorized into the total fixed costs (TFC), total variable costs (TVC), and total costs (TC) as described in previous reports (24, 25). On the other hand, return parameters, including total returns (TR) from chick sale equals kg price (20 LE in May 2017) × body weight and net profit (total returns minus total costs), were calculated. Furthermore, the efficiency of feed additives in the form of collective measures of efficiency was measured according to previous studies (26, 27).

### Statistical Analysis

Data were analyzed by the one-way analysis of variance by the GLM procedure in the SPSS (version 25; IBM Corp., Armonk, NY) statistical software package. Shapiro–Wilk's test was used to calculate the normality, and Hortly's test was used to calculate the homogeneity. The least significance difference test was used to separate significant means (28). The group means were compared using Duncan's multiple range tests (29). The results were reported as mean ± SE (standard error). A *P*-value of <0.05 was considered statistically significant.

## Results and Discussion

The total phenols, flavonoids, and radical scavenging activity of LLE are shown in Table 2. The quantity of extract yield (%) means that every 100 g of raw LLE produces about 24.33 g of dried leek leaf extract. The total phenols and flavonoids of LLE are as high as other plants, and radical scavenging activity is also high. This result helped prompt manner and strong improvement in the poultry growth and serum biochemical parameters.

**Table 2:** Analyses of leek leaf extract.

**Parameters**	**Leek leaf extract (LLE)**
Quantity of extract yield, %	24.33
Total phenolic content	223 mg GAE^*^/g extract powder
Total flavonoid content	65 mg RE^**^/g extract powder
Radical scavenging activity (Inhibition percent, %) at concentration 10 μg/mL	15.00

* *GAE–mg gallic acid equivalents*.

** *RE–mg rutin equivalents*.

Few studies have reported the beneficial effects of LLE as growth promoters in poultry feed. Leek is closely related to garlic and onion, belonging to the same family. LLE has higher total phenolic compounds in the form of organo-sulfur compounds with antioxidant capacity compared with that of garlic and green onion, considering the scavenging or preventive capacity against superoxide anion, hydroxyl, and peroxyl radicals that exert diverse pharmacological functions (4, 30). Also, there is a significant correlation between the total antioxidant capacity and phenolic contents, indicating that the phenolic contents are the dominant antioxidant constituents of LLE (31). LLE contains several biologically active compounds such as ellagic acid, phenols, flavonoids, and tannins that have high antioxidant and anti-inflammatory effects on the intestinal tract (32).

The effect of LLE supplementation in Hubbard diets on productive performance is given in Table 3. LLE-supplemented diets had a significant (*P* = 0.001) effect on the productive performance parameters (FBW, BWG, and total FI) compared with the control. Broilers that fed on the diet containing 0.2% LLE had the heaviest final BW and highest BWG compared with the broilers fed on other LLE treatments. Broilers fed on LLE-supplemented diets showed a significant (*P* = 0.001) increase in the final body weight, BWG, and total FI, while FCR was significantly (*P* = 0.038) improved in the 0.2% LLE supplementation group compared with the control group. Broilers that were fed on a diet contained 0.20% LLE had the heaviest final BW, highest BWG, and best FCR.

**Table 3:** Effect of diets supplemented with leek leaf extract on the overall performance of broiler chicks (means ± SE).

**Trait studied**	**Experimental diets**					***P*-value**
	**Control**	**Leek (kurrat) leaf extract in diets**				
		**0.05%**	**0.10%**	**0.15%**	**0.20%**	
IBW, g	40.20 ± 0.11	40.20 ± 0.05	40.40 ± 0.08	40.37 ± 0.08	40.27 ± 0.04	0.227
FBW, g	1950.20 ± 3.04^d^	2199.99 ± 9.87^c^	2286.50 ± 16.36^b^	2300.50 ± 1.69^b^	2350.46 ± 6.26^a^	0.001
BWG, g	1910.00 ± 3.01^d^	2159.79 ± 9.87^c^	2246.10 ± 16.42^b^	2260.13 ± 1.75^b^	2310.20 ± 6.23^a^	0.001
Total FI, g	3413.07 ± 7.57^c^	3790.67 ± 5.92^b^	3959.90 ± 4.25^a^	3959.53 ± 3.70^a^	3973.80 ± 5.97^a^	0.001
FCR	1.79 ± 0.004^a^	1.76 ± 0.024^a^^b^	1.76 ± 0.011^a^^b^	1.75 ± 0.002^a^^b^	1.72 ± 0.003^b^	0.038

abcd *Means within the same row carrying different superscripts were significantly different at (P ≤ 0.05). IBW, initial body weight; FBW, final body weight; BWG, body weight gain; FI, feed intake; FCR, feed conversion ratio*.

*Number of observation (n) = 50 in each group*.

The diets containing LLE showed a positive effect on the growth performance of the broilers and are in accordance with the previous reports. The performance of broilers was positively affected by the dietary supplementation of 0.75% and 1% of leek powder for 5 weeks according to previous reports (33), and Egyptian leek leaf powder as an unconventional feed at 4, 6, 8, and 10% significantly increased the final body weight, BWG, and total FI of broilers compared with the control group as described earlier (34). Jo et al. (35) stated that broilers fed on diets containing 100 ppm garlic extract showed an improved growth rate during the period of 1–35 days; additionally, the aqueous extract of 1 and 2% of garlic significantly increased the body weight of broilers from 7 to 35 days of age compared with the negative and positive ciprofloxacin controls, and 1% supplementation level showed the highest weight gain and FCR at day 35 as described earlier (36). Goodarzi et al. (37) reported that broilers fed on diets supplemented with 30 g onion/kg diet had a significant increase in the body weights on day 42. Chickens showed a significant increase (*P* <0.05) in average daily feed intake during the grower period and an experimental period of 42 days compared with the control group and a group fed on diet supplemented with an antibiotic. Similarly, chicks fed on diets supplemented with 0.3 and 0.5% onion extract for 35 days showed an increase in BW and BWG compared with those fed on an un-supplemented diet, as reported before (38). In contrast to our results, FI was not affected by the supplementation of garlic extract (36) or onion extract (38) in broiler chicks, as reported previously. The beneficial effects of LLE on the growth performance and FI could be attributed to its high content of polyphenols which increased food and calorie intake (39), high antibacterial activity due to dialkylpolysulfide (40), and increased villi height (33).

Carcass dressing was significantly (*P* = 0.022) increased in LLE-fed groups compared with the control group. The liver percentage was significantly (*P* = 0.041) increased in the group fed on the diet supplemented with 0.2% LLE, and a non-significant increase was observed in the other LLE-supplemented groups compared with the control group (Table 4). No significant difference in the heart (*P* = 0.943), intestine (*P* = 0.504), spleen (*P* = 0.065), and stomach (*P* = 0.637) was observed among different experimental groups compared with the control group.

**Table 4:** Effect of diets supplemented with leek leaf extract on carcass traits relative to the live weight of broiler chickens (means ± SE).

**Parameters**	**Experimental diets**					***P*-value**
	**Control**	**Leek leaf extract in diets**				
		**0.05%**	**0.10%**	**0.15%**	**0.20%**	
Dressing %	66.82 ± 0.71^b^	70.56 ± 0.22^a^^b^	72.52 ± 0.52^a^	72.07 ± 0.96^a^	73.32 ± 1.55^a^	0.022
Liver %	2.88 ± 0.16^b^	3.19 ± 0.24^a^^b^	3.30 ± 0.08^a^^b^	3.29 ± 0.11^a^^b^	3.52 ± 0.05^a^	0.041
Heart %	0.74 ± 0.19	0.76 ± 0.06	0.74 ± 0.01	0.68 ± 0.08	0.80 ± 0.09	0.943
Stomach %	3.95 ± 0.48	4.20 ± 0.38	4.34 ± 0.05	4.32 ± 0.14	4.32 ± 0.18	0.637
Intestine %	5.88 ± 0.29	5.77 ± 0.11	5.74 ± 0.13	5.43 ± 0.14	5.68 ± 0.04	0.504
Spleen %	0.37 ± 0.013	0.38 ± 0.012	0.35 ± 0.007	0.38 ± 0.007	0.38 ± 0.010	0.065

ab *Means within the same row carrying different superscripts were significantly different at (P ≤ 0.05)*.

*Number of observation (n) = 5 in each group*.

Also, the broilers that fed on garlic extract 40 or 60 ppm/kg diet from day 13 to 47 (41) and on 0.3 or 0.5% of onion extract for 5 weeks (38) showed an increase in carcass dressing % similar to our results. In contrast to our result, others reported that broilers that fed on diets containing 25, 50, and 100 mg of garlic or onion for 21 days had a non-significant effect on the carcass dressing (42). Diets containing garlic (36, 43) or onion extracts (38) showed a non-significant increase in the weights of different visceral organs of broilers, similar to the present study. An increase in dressing %, especially in the LLE fed group, could be attributed to the effect of LLE on final body weight that may have reflected in the dressing weight.

Biochemical parameters of blood are the valid indicators of health (physiological and nutritional) status of the broilers. The effect of LLE-containing diets on the serum metabolites of the broilers is given in Table 5. There were no significant changes in total protein (*P* = 0.692), albumin (*P* = 0.748), globulin (*P* = 0.163), and liver enzymatic activity (ALT and AST) (*P* = 0.059). Also, no significant changes in serum creatinine (*P* = 0.547), urea (*P* = 0.609), and uric acid (*P* = 0.999) were observed in the broilers fed on LLE compared with the control group. These results are similar to previous reports (34) on liver enzymes (ALT and AST), creatinine, urea, total protein, albumin, and globulin of broilers fed on diets supplemented with 2, 4, 6, 8, and 10% of leek leaf powder. We found that the effect of diets supplemented with LLE was non-significant on the serum total protein and albumin levels and is in accordance with other studies performed on the dietary supplementation of allicin (44). Serum AST and ALT are considered important parameters to evaluate the effect of unconventional feed stuff or new feed additives on broilers (45, 46). We found no significant changes in the serum AST and ALT levels of broilers that fed on LLE-containing diets. Similarly, broilers fed on diets containing garlic extracts (36) and onion extracts (38) showed no significant effect on AST and ALT levels, as reported previously. Supplementation of onion juice of various concentrations had a non-significant effect on the levels of serum urea, uric acid, and creatinine in rats, as described previously (47).

**Table 5:** Effect of diets supplemented with leek leaf extract on some serum biochemical analysis of broiler chickens (means ± SE).

**Parameters**	**Experimental diets**					***P*-value**
	**Control**	**Leek leaf extract in diets**				
		**0.05%**	**0.10%**	**0.15%**	**0.20%**	
**Effect leek leaf extract on liver function**						
Total protein, g/dl	4.27 ± 0.92	4.30 ± 0.58	4.17 ± 0.20	4.20 ± 0.05	4.19 ± 0.20	0.692
Albumin, g/dl	1.70 ± 0.02	1.69 ± 0.02	1.71 ± 0.01	1.70 ± 0.01	1.70 ± 0.01	0.748
Globulin, g/dl	2.57 ± 0.01	2.61 ± 0.12	2.46 ± 0.05	2.50 ± 0.08	2.49 ± 0.06	0.163
AST, IU/dl	39.00 ± 1.52	39.33 ± 2.40	37.33 ± 1.45	40.33 ± 2.28	36.67 ± 0.33	0.059
ALT, IU/dl	23.67 ± 1.33	24.33 ± 0.88	22.00 ± 1.73	23.67 ± 1.76	19.33 ± 1.85	0.059
**Effect leek leaf extract on kidney function**						
Creatinine, mg/dl	0.37 ± 0.03	0.39 ± 0.01	0.40 ± 0.02	0.41 ± 0.02	0.41 ± 0.02	0.547
Urea, mg/dl	2.65 ± 0.003	2.66 ± 0.005	2.65 ± 0.006	2.64 ± 0.006	2.66 ± 0.008	0.609
Uric acid, mg/dl	3.21 ± 0.044	3.20 ± 0.011	3.22 ± 0.012	3.23 ± 0.120	3.22 ± 0.017	0.999
**Effect leek leaf extract on glucose**						
Glucose, mg/dl	119.93 ± 1.35^a^	89.17 ± 1.68^b^	88.93 ± 1.17^b^	80.47 ± 0.83^c^	80.63 ± 1.16^c^	0.001
**Effect leek leaf extract on lipid profile**						
Cholesterol, mg/dl	153.33 ± 2.09^a^	113.07 ± 1.62^b^	105.97 ± 1.95^b^^c^	101.43 ± 0.32^c^	102.23 ± 0.89^c^	0.001
Triglyceride, mg/dl	130.37 ± 1.13^a^	102.90 ± 1.30^b^	102.27 ± 0.66^b^	94.93 ± 0.37^c^	94.17 ± 0.84^c^	0.001
HDL, mg/dl	68.40 ± 0.77^c^	88.00 ± 1.30^b^	87.20 ± 0.34^b^	104.47 ± 2.48^a^	103.00 ± 1.37^a^	0.001
LDL, mg/dl	195.23 ± 0.48^a^	114.70 ± 3.25^b^	111.50 ± 4.05^b^	107.70 ± 1.27^b^	110.23 ± 1.50^b^	0.001

abc *Means within the same row carrying different superscripts were significantly different at (P ≤ 0.05)*.

*Number of observation (n) = 5 in each group. AST, Serum aspartate-aminotransferase; ALT, Alanine aminotransferase; HDL, High density lipoprotein; LDL, Low density lipoprotein*.

Interestingly, broilers fed on LLE-supplemented diets had a significant (*P* = 0.001) reduction in serum glucose level compared with the control group (Table 5). Our results are in accordance with other studies (33, 34) experimented on the blood glucose levels of broilers. Also, a reduction in blood glucose level in diabetic rats fed diets containing leek (*Allium Ampeloprasum*) for 1 month (5) and diabetic rabbits fed diets containing an aqueous extract of onions has been reported (48). The hypoglycemic effects of leek may be attributed to allyl-propyl-disulfide compounds present in it that compete with the insulin for metabolism, thus increasing the free insulin. Other mechanisms postulated are sulfur-containing compounds, including dialkyl disulfides, and their oxidized thiols can trap electrons from the body to act as antioxidants or phenolic acids produced antioxidant activity (48, 49).

Furthermore, broilers fed on the diets containing LLE showed a significant (*P* = 0.001) decrease in serum levels of total cholesterol, triglyceride, and LDL and a significant (*P* = 0.001) increase in HDL levels compared with the control group (Table 5). Similarly, serum levels of total cholesterol, triglyceride, and LDL cholesterol were significantly decreased in rabbits fed diets containing leek extract (6) and broilers that fed on diets containing leek powder as reported previously by Kamali et al. and Mahmoud et al. (33, 34) and broilers that fed on diets containing onion extract (38). The hypocholesterolemic and hypolipidemic effects of leek may be because of high levels of sulfur-containing compounds such as S-methylcysteine sulfoxide (50). Contrary to our results, Adjei et al. (44) showed no significant reduction in LDL, VLDL, total cholesterol, and triglycerides levels by dietary supplementation with varying levels of allicin in broilers.

The effect of diets containing LLE on economic measures of broilers is given in Table 6. We found a significant (*P* = 0.001) increase in feed costs, TVC, TC, return and net profit, and collective economic efficiency measures in all the groups fed on the diets containing LLE compared with the control group. Broilers fed on the diets containing 0.2% LLE showed the highest return, net profit values, and net profit/total cost with the lowest values for the control group.

**Table 6:** Effect of diets supplemented with leek leaf extract on economic measures of broiler chickens (means ± SEM).

**Trait studied**	**Experimental diets**					***P*-value**
	**Control**	**Leek leaf extract in diets**				
		**0.05%**	**0.10%**	**0.15%**	**0.20%**	
**Cost parameters**						
Feed costs	21.28 ± 0.05^d^	23.95 ± 0.23^c^	25.46 ± 0.03^b^	25.54 ± 0.02^a^^b^	25.86 ± 0.04^a^	0.001
Total variable costs	21.48 ± 0.05^d^	24.16 ± 0.23^c^	25.47 ± 0.03^b^	25.74 ± 0.02^b^	26.07 ± 0.04^a^	0.001
Total cost	44.40 ± 0.05^d^	47.08 ± 0.23^c^	48.39 ± 0.02^b^	48.66 ± 0.03^b^	48.99 ± 0.04^a^	0.001
**Return parameters**						
Total returns	44.85 ± 0.07^d^	50.60 ± 0.23^c^	52.59 ± 0.37^b^	52.91 ± 0.38^b^	54.06 ± 0.14^a^	0.001
Net profit	0.45 ± 0.10^c^	3.52 ± 0.45^b^	4.20 ± 0.35^a^^b^	4.25 ± 0.05^a^^b^	5.07 ± 0.11^a^	0.001
**Collective measures of feed additive efficiency**						
Net profit / total cost %.	1.01 ± 0.22^c^	7.49 ± 1.00^b^	8.69 ± 0.73^a^^b^	8.73 ± 0.08^a^^b^	10.35 ± 0.22^a^	0.001

abcd *Means within the same row carrying different superscripts were significantly different at (P ≤ 0.05)*.

## Conclusion

Dietary supplementation with leek (*Allium ampeloprasum var. kurrat*) leaf extract in broilers provided a safe growth-promoting effect without any adverse effects in terms of biochemical parameters and carcass quality. Furthermore, the diets containing LLE can improve economic efficiency by increasing the return and net profit. Finally, LLE can act as an eco-friendly alternative for antibiotic growth promoters in the poultry industry.

## Data Availability Statement

The original contributions generated for this study are included in the article/supplementary materials, further inquiries can be directed to the corresponding author/s.

## Ethics Statement

The animal study was reviewed and approved by Institutional Animal Care and Use Committee of Zagazig University, Egypt (ZUIACUC−2019), and all animal experiments were performed following recommendations described in The Guide for the Care and Use of Laboratory Animals in scientific investigations.

## Author Contributions

MB, AO, and HA-k: design of the experiment. MB, AO, RA, and MA: methodology, data collection, and analysis. HA-k, MB, AO, RA, and MA: writing of the manuscript. All authors have read and approved the manuscript.

## Conflict of Interest

The authors declare that the research was conducted in the absence of any commercial or financial relationships that could be construed as a potential conflict of interest.
